# Targeted delivery of CRISPR/Cas9 to prostate cancer by modified gRNA using a flexible aptamer-cationic liposome

**DOI:** 10.18632/oncotarget.14072

**Published:** 2016-12-21

**Authors:** Shuai Zhen, Yoichiro Takahashi, Shunichi Narita, Yi-Chen Yang, Xu Li

**Affiliations:** ^1^ Center for Translational Medicine, The First Affiliated Hospital of Xi’an Jiaotong University, P.R. China; ^2^ Key Laboratory for Tumor Precision Medicine of Shaanxi Province, The First Affiliated Hospital of Xi’an Jiaotong University, P.R. China; ^3^ Center for iPS Cell Research and Application (CiRA), Kyoto University, Kyoto, Japan; ^4^ Tsinghua University, School of Life Sciences, Beijing, China

**Keywords:** CRISPR/Cas9 delivery, aptamer-liposome chimera, prostate cancer, prostate-specific membrane antigen (PSMA), polo-like kinase 1 (PLK1)

## Abstract

The potent ability of CRISPR/Cas9 system to inhibit the expression of targeted gene is being exploited as a new class of therapeutics for a variety of diseases. However, the efficient and safe delivery of CRISPR/Cas9 into specific cell populations is still the principal challenge in the clinical development of CRISPR/Cas9 therapeutics. In this study, a flexible aptamer-liposome-CRISPR/Cas9 chimera was designed to combine efficient delivery and increased flexibility. Our chimera incorporated an RNA aptamer that specifically binds prostate cancer cells expressing the prostate-specific membrane antigen as a ligand. Cationic liposomes were linked to aptamers by the post-insertion method and were used to deliver therapeutic CRISPR/Cas9 that target the survival gene, polo-like kinase 1, in tumor cells. We demonstrate that the aptamer-liposome-CRISPR/Cas9 chimeras had a significant cell-type binding specificity and a remarkable gene silencing effect *in vitro*. Furthermore, silencing promoted a conspicuous regression of prostate cancer *in vivo*. Importantly, the approach described here provides a universal means of cell type–specific CRISPR/Cas9 delivery, which is a critical goal for the widespread therapeutic applicability of CRISPR/Cas9 or other nucleic acid drugs.

## INTRODUCTION

Clustered regularly-interspaced short palindromic repeats (CRISPRs) is first identified in bacteria and archaea as an adaptive immune response via encoding CRISPR RNAs to guide CRISPR-associated (Cas) nucleases to invading genetic material [1, 2]. Later, the potential of Cas9 from the type II CRISPR system of S. pyogenes in RNA-guided precise genome editing is found in eukaryotic organisms [3–6], which is rapidly proving to be an efficient DNA-targeting strategy for multiple applications including gene expression modulation [7] and imaging [8]. Cas9 uses gRNA to locate site-specific DNA sequence, form base pairs and introduce a double strand break. Altering the first 20 nt of the gRNA can direct Cas9 nuclease to specific sites, CRISPR/Cas9 therefore provides uniquely flexible and accessible means for genome editing. In view of its specificity, efficiency, simplicity and versatility, CRISPR/Cas9 system shows great promise for the treatment of many diseases, such as genetic disorders, cancer, and virus infection [9–11]. Like other biological drugs, CRISPR/Cas9-based therapeutics often require a delivery vehicle to transport them to the targeted tissues or cells. Thus, the advancement of numerous CRISPR/Cas9 drugs has relied on the development of CRISPR/Cas9 carriers [12]. However, the efficient *in vivo* delivery of CRISPR/Cas9 remains a major challenge, thus greatly restrains its clinic application [12]. Particularly, targeted delivery techniques for CRISPR/Cas9 into specific cell populations or tissues is highly desirable for improving the safety and efficacy of CRISPR/Cas9- based therapeutics.

The development of targeted delivery has progressed rapidly in recent years. Two indispensable parts are required for an ideal targeted delivery system: (i) a safe vehicle, which can protect RNA from nuclease degradation in the bloodstream; (ii) a targeting moiety/ligand, which can specifically recognize the receptor and effectively escort cargo into a selective tissue or cell. Thus, a targeting ligand with high specificity and affinity to a cellular receptor is a crucial factor in establishing a targeted CRISPR/Cas9 delivery system [13].

More recently, nucleic acid-based aptamers have been described as non-protein-based alternatives to antibodies, and thus possess the potential as targeting agents for the delivery of cargoes [14]. A new concept dubbed as ‘escort aptamers’ by Hicke and Stephens [15] develops a new field of aptamer functionality. The nucleic acid composition endows escort aptamers with unique features including high sensitivity and specificity, small size, low immunogenicity, and convenience of *in vitro* selection which enable escort aptamers applicable in various molecular targeting [16].

Quite a few aptamers have been successfully adapted for the targeted delivery of active therapeutics *in vitro* and *in vivo* via specific cell surface receptors. For example, cell-internalizing aptamers have been applied to specifically deliver siRNAs into target cells [17]. The best characterized and well-established aptamers for molecules delivery are the prostate-specific membrane antigen (PSMA) aptamers [18]. It has been reported that a gp120 aptamer-siRNA chimera successfully delivers siRNAs targeting the HIV-1 *tat/rev* common exon in both cell and mouse models [19, 20]. Additionally, aptamer-siRNA conjugates is able to deliver siRNAs into tumor cells [18, 21, 22]. However, the targeted delivery of CRISPR/Cas9 system has not been reported yet. In the present study, we intend to develop a universal system that combines efficient delivery and modified flexibility. An aptamer-liposome-CRISPR/Cas9 chimera-based approach is described for specific delivery of gRNA. The RNA aptamer A10 is reported to deliver therapeutic CRISPR/Cas9-gRNA targeting polo-like kinase 1, a pro-survival gene overexpressed in most human tumors into prostate cancer cells via specifically binding to the cell-surface receptor PSMA. We demonstrate that the aptamer-liposome- CRISPR/Cas9 chimeras not only had a significant cell-type specificity in binding and a remarkable gene silencing effect *in vitro*, but also a conspicuous tumor regression *in vivo*. Notably, the approach introduced here provides a means of cell type-specific CRISPR/Cas9 delivery to realize the therapeutic applicability of CRISPR/Cas9.

## RESULTS

### Preparation and characterization of A10-liposome-siRNA chimeras

**Figure 1:**
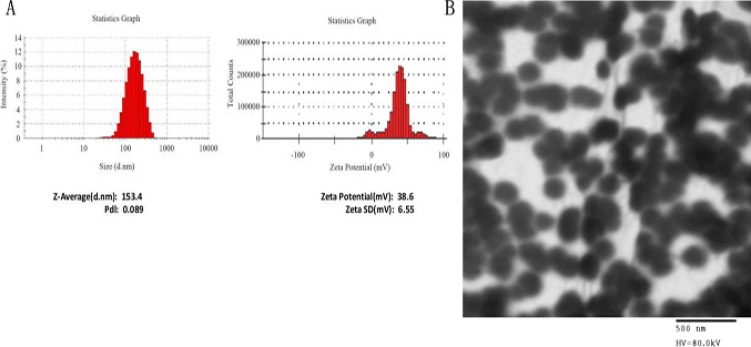
Characterization of A10-liposome-CRISPR/Cas9 chimeras (**A**) Size and Zeta potential of A10-liposome- CRISPR/Cas9 chimeras. The liposome-CRISPR/Cas9 chimeras were determined using a Nano Zetasizer (Malvern Instrument Ltd.) in HEPES buffer. (**B**) Transmission electron photomicrograms of cationic liposomes. Particles were imaged using an accelerating voltage of 80 kV (magnification: × 60, 000).

A10-liposome-CRISPR/Cas9 chimeras were prepared using a post-insertion method by mixing poly (ethylene glycol)-grafted 1,2-distearoyl-sn-glycero-3-phosphatidylethanolamine (DSPE-PEG)-modified A10 and liposome- CRISPR/Cas9 complexes at room temperature. The liposome/gRNA ratio was 300:1, and the weight ratio of protamine/gRNA was 0.75. As shown in Figure 1, the liposome- CRISPR/Cas9 chimeras were of the appropriate particle size and zeta potential. The average particle size of A10-liposome- CRISPR/Cas9 chimeras as determined by dynamic light scattering was ∼150 nm (Figure 1A, left panel). Zeta potential is a key factor in the stability of a colloidal dispersion. Weak negative charges of ∼40 mV were observed for the prepared chimeras (Figure 1A, right panel). TEM (Transmission Electron Microscope) (Figure 1B) verified the size and showed that the A10-liposome-siRNA chimeras consist of uniform particles.

### Modified A10 binds PSMA-expressing cells

**Figure 2:**
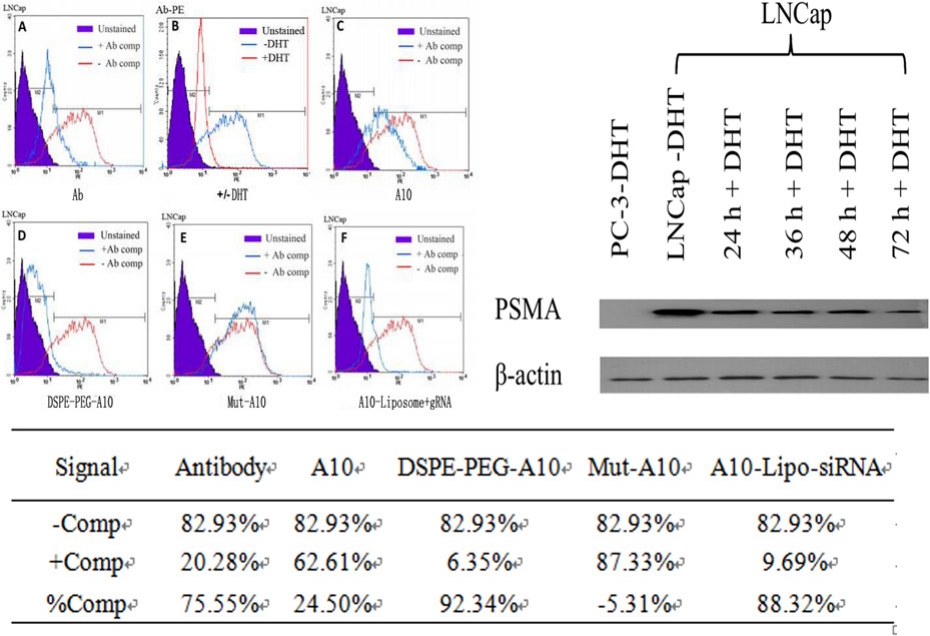
Cell surface competition assay of modified A10 binding to PSMA PE-labeled PSMA antibody was bound to LNCap cells (red), and then competition assays were performed with unlabeled PSMA antibody as a positive control (**A**) or with different modified A10 conjugations (antibody competitors) (**C**–**F**) (blue). Cell surface competition was assessed by flow cytometry. As a control for specificity of binding, DHT treatment, which is known to reduce total PSMA protein expression, was shown to reduce the binding of PE-labeled PSMA antibody (**B**). Total PSMA protein expression in LNcap or PC-3 cells was determined by Western Blotting. The percentage of PE positive cells following the addition of each competitor is listed in the table.

To determine the binding of the A10-liposome- CRISPR/Cas9 chimeras to PSMA on prostate cancer cells, we performed competition assays against PE-labeled PSMA antibody bound to LNCap cells. LNCap cells were selected because PSMA specifically existed on the surface of LNCap rather than PC-3 prostate cancer cell line [23] (Total PSMA protein expression in LNcap and PC-3 cells was determined by Western Blotting). As a control, staining with PE-labeled PSMA antibody was shown to be competed competitively by unlabeled PSMA antibody (Figure 2A). As an additional control, we tested binding of PE-labeled PSMA antibody on LNCap cells pretreated with the 5-a-dihydrotestosterone (DHT) which could reduce the expression of PSMA. As expected, the binding of PE-labeled PSMA antibody was reduced following DHT treatment, verifying the correlation of binding to PSMA on the cells surface (Figure 2B). These results confirm the specificity and quantitative ability of this assay.

To assess the ability of the aptamer and modified aptamer conjugates to bind to PSMA on the surface of prostate cancer cells, PE-labeled PSMA antibody was bound to LNCap cells, and then the competition ability of the aptamer complexes was tested. These aptamer complexes included A10 without modification, DSPE-PEG linked A10, mut-A10 (a non-targeting A10 RNA sequence with two mutated bases) and A10-liposome-CRISPR/Cas9 chimeras. Bindings of both A10 (Figure 2C) and DSPE-PEG linked A10 (Figure 2D) showed significant competition relative to PE-labeled PSMA antibody, while mut-A10 (Figure 2E) was unable to compete with the PSMA antibody. This demonstrates that A10 aptamer with or without DSPE-PEG modification has the ability to specifically bind to PSMA on LNCap cells, and this binding is dependent on the sequence-specificity of A10 RNA. Binding of DSPE-PEG linked A10 (Figure 2D) and A10-liposome- CRISPR/Cas9 chimeras (Figure 2F) showed similar competition against binding of PE-labeled PSMA antibody, indicating that aptamer-containing chimeras retain their ability of binding PSMA on the surface of LNCap cells.

### Assessment of liposome- CRISPR/Cas9 chimera activity by *in vitro* gene knockdown assay

**Figure 3:**
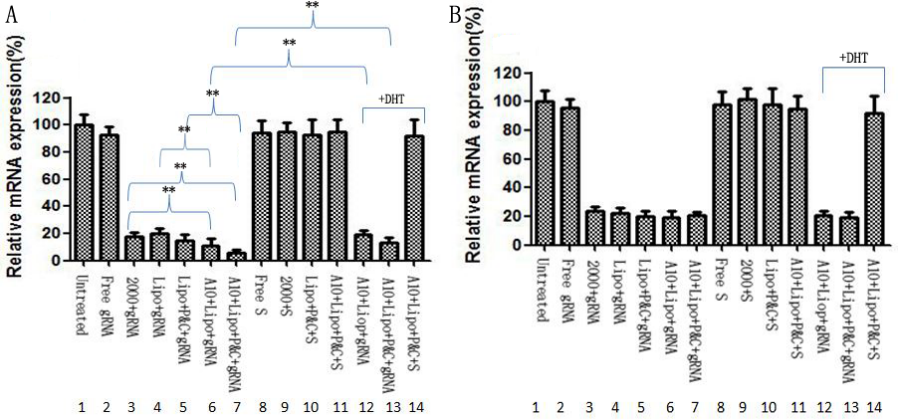
mRNA silencing in LNCap cells treated with different liposome chimeras LNCap cells (**A**) or PC-3 cells (**B**) were transfected with 400 nM free CRISPR/Cas9 (panel 2), CRISPR/Cas9 transfected with Lipofectamine-2000 (panel 3, as positive control), liposome-CRISPR/Cas9 chimeras (panel 4), liposome-CRISPR/Cas9 chimeras with protamine and calf thymus (panel 5), A10-liposome-CRISPR/Cas9 chimeras (panel 6), A10-liposome-CRISPR/Cas9 chimeras with protamine and calf thymus (panel 7). As contrast, the silencing effect was also determined by scrambled CRISPR/Cas9 alone (panel 8), or formulated in Lipofectamine-2000 (panel 9), or in liposome-chimeras with protamine and calf thymus (panel 10), or in A10-liposome chimeras (panel 11). PLK1 mRNA expression was assessed by RT-PCR. To further verify that silencing by liposome-CRISPR/Cas9 chimeras is dependent on PSMA, LNcap or PC-3 cells were incubated with 2 nM DHT for 48 h before the addition of chimeras. Then, cells were treated with A10-liposome- CRISPR/Cas9 chimeras (panel 12), or A10-liposome-CRISPR/Cas9 chimeras with protamine and calf thymus (panel 13), or A10-liposome- scrambled CRISPR/Cas9 chimeras with protamine and calf thymus (panel 14). Results represent the mean + SD of PLK1 mRNA expression for 3 replicates standardized to GAPDH mRNA expression and normalized to an average of 100% in the untreated cells. 2000, lipofectamine 2000; P&C, protamine and calf thymus; Lipo, liposome; S, scramble gRNA *p < 0.05; **p < 0.01.

To demonstrate the biological activity of liposome-CRISPR/Cas9 chimeras, we analyzed PLK1 mRNA levels by RT-PCR in cells after treatment with different formulations of CRISPR/Cas9 reagents (Figure 3). Free PLK1 CRISPR/Cas9 (Figure 3A, lane 2) had little effect due to the poor cellular bioavailability of its negative charge. Liposome chimeras containing protamine and calf thymus DNA (Figure 3A, lane 5, 7) down-regulated PLK1 mRNA, better than the corresponding result of liposome- CRISPR/Cas9 chimeras without protamine and calf thymus DNA (Figure 3A, lane 4, 6), suggesting that protamine and calf thymus can partly improve the transfection efficiency. It also can be seen that, even without A10, the liposome-CRISPR/Cas9 chimeras (Figure 3A, lane 5) we described had the same effect of lipofectamine-2000 (Figure 3A, lane 3), an acknowledged commercial transfection reagent. Further, with the attendance of A10, the liposome-CRISPR/Cas9 chimeras (Figure 3A, lane 7) down-regulated 63% PLK1 mRNA, significantly better than chimeras without A10 (Figure 3A, lane 5) (*P* < 0.01). In contract to LNCap cells, PLK1 mRNA knockdown in PC-3 cells had no correlations with chimeras’ formulation, only depended on CRISPR/Cas9 targeting (Figure 3B). These results demonstrate that A10 aptamer greatly improves the transfection efficiency.

To verify that silencing effect of liposome-CRISPR/Cas9 chimeras was dependent on PSMA, LNcap cells were incubated with 2 nM DHT for 48 h before the addition of chimeras (Figure 3A, lane 8, 9). In the presence of DHT, PLK1 gene silencing was significantly less effective (Figure 3A, lane 6, 7). In PC-3 cells, there was no difference before (Figure 3B, lane 6, 7, 11) and after DHT addition (Figure 3B, lane 12, 13, 14). These data indicate that the gene silencing effect of this novel delivery system is dependent on cell surface expression status of PSMA.

### Assessment of liposome-CRISPR/Cas9 chimera activity in the intracellular uptake of CRISPR/Cas9 *in vitro*

**Figure 4:**
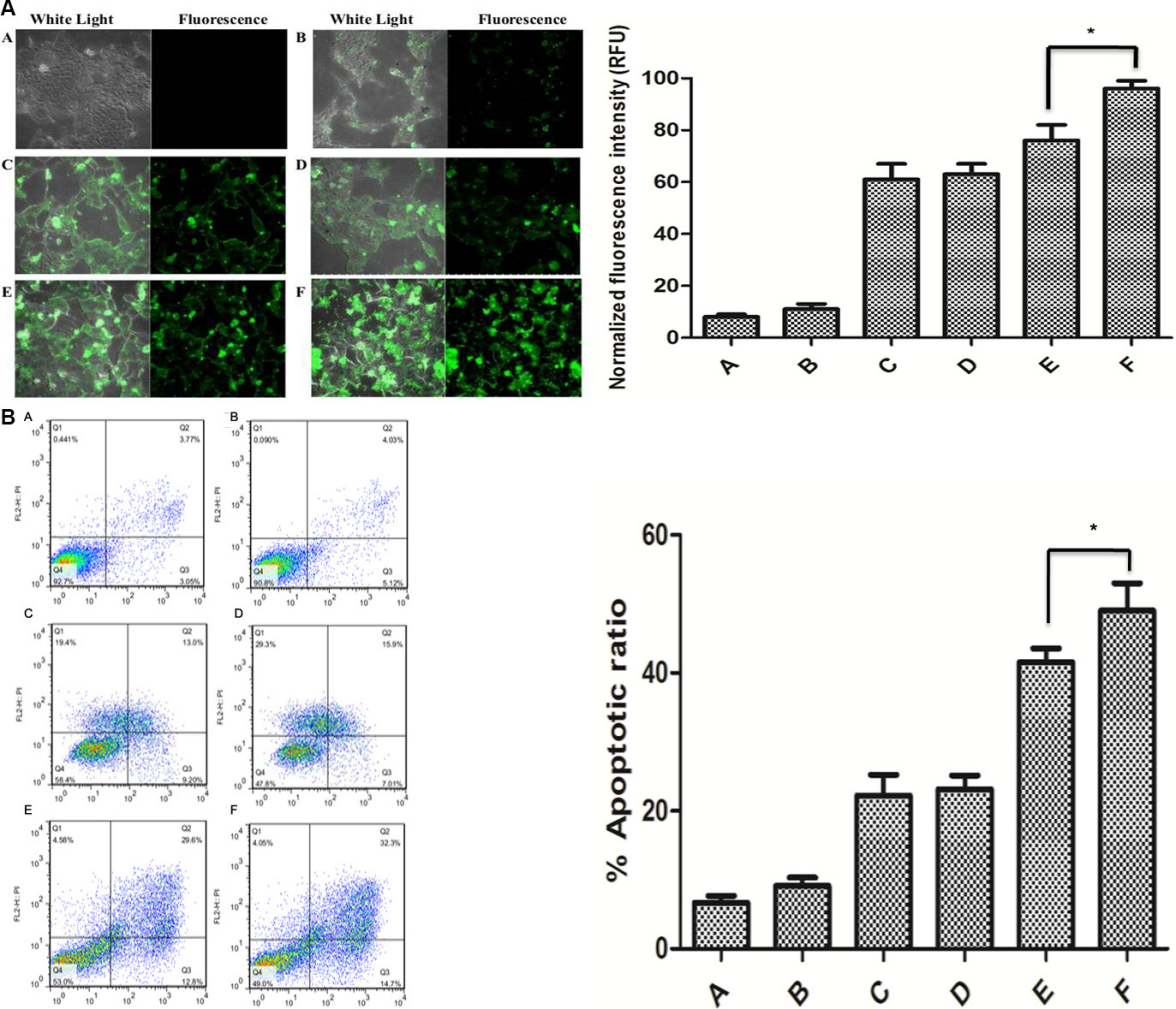
Intracellular uptake of 5′-FAM labeled CRISPR/Cas9 in LNCap cells and analysis of cell apoptosis after treatment with in different liposome chimeras (**A**) Cells were treated with different liposome-CRISPR/Cas9 formulations at 37°C for 4 h. Cells were rinsed, fixed, and intracellular uptake of the CRISPR/Cas9 was imaged fluorescent microscopy. (A) no CRISPR/Cas9; (**B**) gRNA empty vector-CRISPR/Cas9; (**C**) Lipofectamine-2000 + CRISPR/Cas9; (**D**) Liposome + CRISPR/Cas9; (**E**) Liposome + Protamine + calf thymus DNA + CRISPR/Cas9; (**F**) A10-Liposome + Protamine + calf thymus DNA + CRISPR/Cas9. Magnification = 400×. (B) Cell apoptosis following exposure to liposome chimeras.

To verify that the increased gene silencing mediated by the A10-liposome- CRISPR/Cas9 chimeras and protamine + calf thymus DNA was due to increased delivery, we performed cellular uptake assays. The uptake of fluorescently labeled CRISPR/Cas9 in the cytoplasm of cells was observed by confocal microscopy. As shown in Figure 4A, the uptake of CRISPR/Cas9 formulated with liposome chimeras with protamine and calf thymus DNA (Figure 4E) was much greater than with liposome (Figure 4D) or lipofectamine-2000 (Figure 4C). Furthermore, the fluorescence signal in cells treated with A10-liposome-CRISPR/Cas9 chimeras (Figure 4F) was much stronger than that of cells treated with liposome alone (Figure 4E). Thus, these results indicated that liposome chimeras with A10 aptamer mediated the strongest gene silencing effect due to efficient delivery of CRISPR/Cas9 into LNCap cells.

PLK1 was a valid target in tumors since its inhibition induced apoptosis in proliferating cancer cells [24, 25]. We then examined whether the indicated liposome chimeras either alone or in the presence of CRISPR/Cas9 could induce apoptosis in treated cells, and whether modifications of the liposomes could enhance their pro-apoptotic activity. As shown in Figure 4B, the apoptosis was originated from the downregulation of PLK-1 in LNCap cells.

### Assessment of cell viability following treatment with liposome- CRISPR/Cas9 chimeras

**Figure 5:**
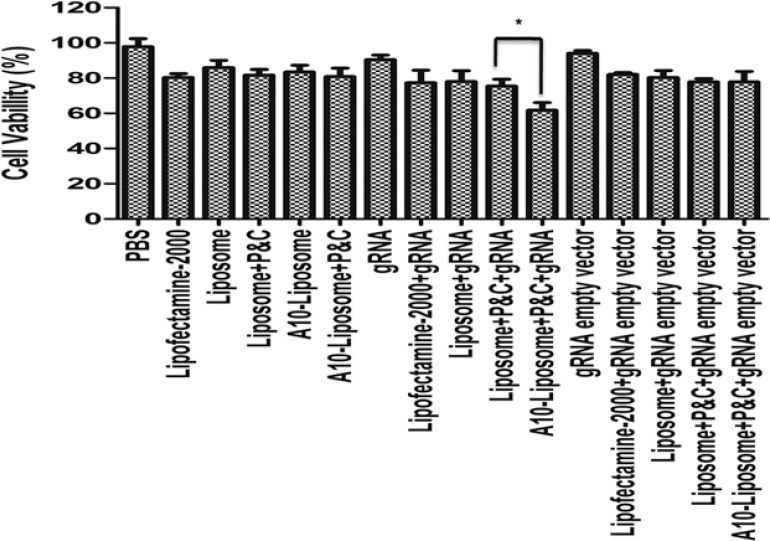
Viability (%) of LNCap cells treated with different liposome chimeras Cells were seeded at 10^5^ cells/mL in a 96-well plate and incubated at 37°C. Percentage of cell viability was determined following 24 h exposed to different liposome chimeras by MTT assay. data represents the percentage of cell viability compared with untreated cells. Data = mean ± SD (*n* = 6). * indicates a significant difference between two groups (*p* < 0.05).

PLK1 is a survival gene that is over-expressed in most human tumors [24, 25]. Therefore, knockdown PLK1 would be expected to reduce the viability of LNCap cells. To assess the cell viability following treatment with liposome chimeras, either alone or in the presence of CRISPR/Cas9, the cytotoxic effects of different liposome formulations were measured by MTT assay (Figure 5). Free CRISPR/Cas9 alone had low toxicity, consistent with its poor ability to knockdown genes without a delivery agent (Figure 3). In the absence of CRISPR/Cas9, our liposome chimera preparations (with or without A10 aptamer) were less toxic than commercial lipofectamine-2000, though the toxicity was increased somewhat with the addition of protamine and calf thymus DNA. In the presence of scrambled CRISPR/Cas9, different formulation groups showed comparative toxicity to the lipofectamine-2000. These results suggest that the liposomes we developed are not inherently cytotoxic. However, the addition of CRISPR/Cas9 targeted PLK1 decreased the cell survival when added with lipofectamine-2000 or liposome, and this effect was greater when protamine and calf thymus DNA was added. The most dramatic decrease in viability was observed for the CRISPR/Cas9 delivered by A10-liposome chimeras with protamine and calf thymus DNA. Thus, the toxicity of the A10-liposome-CRISPR/Cas9 chimeras appears to be specific for the PLK1 CRISPR/Cas9 and correlates with differences in the specific uptake by the different delivery systems.

### A10-liposome-CRISPR/Cas9 chimeras promote tumor regression

**Figure 6:**
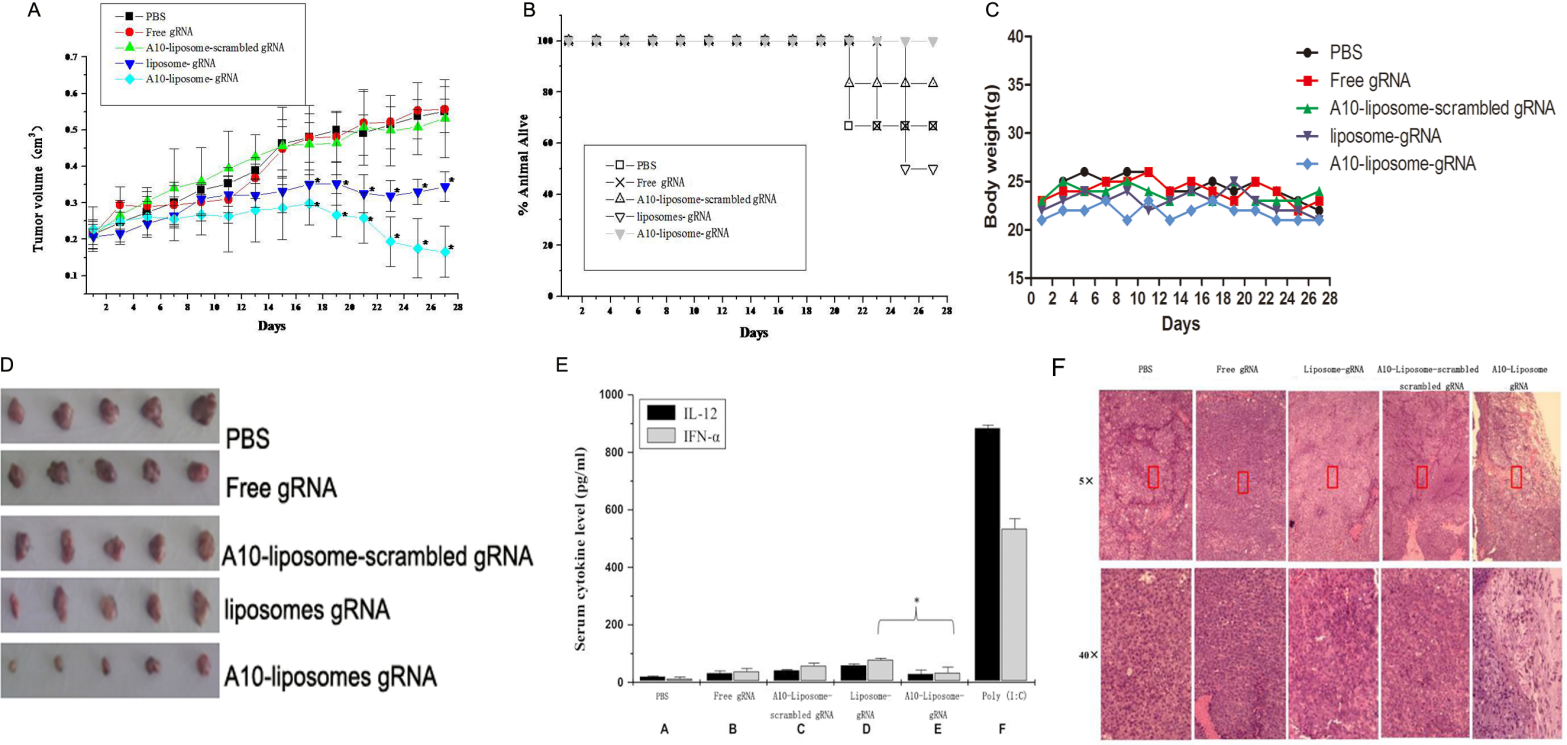
Antitumor activity of A10-liposome-CRISPR/Cas9 chimeras in a xenograft model of prostate cancer (*n* = 8 /group) (**A**) Different CRISPR/Cas9 formulations (Free CRISPR/Cas9, red; liposomes-CRISPR/Cas9 chimera, green; A10-liposome-scrambled CRISPR/Cas9 chimera, dark blue; A10-liposome-CRISPR/Cas9 chimera, light blue) were administered i.v. in a mouse xenograft model bearing PSMA-positive LNCap prostate cancer cells implanted into the shoulder flanks of nude mice. Saline (PBS) treated animals were used as a control (black). Tumors were measured every two days. The mean tumor volumes were analyzed using one-way ANOVA. *p < 0.05 vs the free CRISPR/Cas9 group. (**B**) Death protection for mice after treatment with different CRISPR/Cas9 formulations. The extension of death was analyzed using a sequential chi-square test. (**C**) Mice after treatment with different CRISPR/Cas9 formulations, body weight changes (**D**) Mice were sacrificed and the tumors were isolated. (**E**) Assessment of potential immunostimulatory effects promoted by the delivery system. Serum from mice treated with saline (PBS), free CRISPR/Cas9, A10-liposome-scrambled CRISPR/Cas9, liposome-CRISPR/Cas9, A10-liposome-CRISPR/Cas9 or poly I:C was assessed for levels of IFN-α and IL-12 using ELISA. Data represent means ± SD (*n* = 5) (**F**) Histology of LNCap tumors treated with the various chimeras. Serial sections of formalin-fixed tumors embedded in paraffin were stained with hematoxylin and eosin (H&E) and analyzed at 5× and 40× magnification (boxed region is amplified eight times in the bottom panels).

To determine the efficacy of A10-liposome-CRISPR/Cas9 chimeras *in vivo*, we detected the ability of the A10-liposome-CRISPR/Cas9 chimeras to retard xenograft tumor growth in athymic mice derived from PSMA-positive human prostate cancer cells (LNCap). Mice bearing xenograft tumors larger than 0.2 cm*^3^* were injected *i.v*. (day 0) with PBS, PLK1- CRISPR/Cas9 alone (free CRISPR/Cas9) or chimeric PLK1-CRISPR/Cas9 (A10-liposome-scrambled CRISPR/Cas9, liposome- CRISPR/Cas9, or A10-liposome-CRISPR/Cas9). Tumors were measured every other day (Figure 6A). No obvious difference in tumor volume was observed between the PBS control and the free CRISPR/Cas9 group, showing that CRISPR/Cas9 delivery is necessary for tumor regression. Additionally, the A10-liposome-scrambled CRISPR/Cas9 group did not reduce tumor volume, suggesting specificity for the PLK1 CRISPR/Cas9 target sequence. In contrast, a substantial reduction in tumor volume was observed for both the liposome-CRISPR/Cas9 group and the A10-liposome-CRISPR/Cas9 group. Indeed, from day 13 to day 27 the PBS-treated tumors increased 2.56-fold in volume, whereas the A10-liposome-CRISPR/Cas9 treated tumors reduced 1.66-fold in volume. Compared to the liposome-CRISPR/Cas9 group, the A10-liposome-CRISPR/Cas9 chimeras had a more significant regression (0.40 cm*^3^* to 0.15 cm*^3^*), demonstrating that the regression of tumor volume is improved by the presence of A10. The Chi-square values of the survival rate of the A10-liposome-scramble CRISPR/Cas9 group, liposome-CRISPR/Cas9 group and A10-liposome- CRISPR/Cas9 group were 1.20, 6.00 and 11.58, respectively, only the latter two groups fell within the 99% confidence intervals for statistically significant difference with free CRISPR/Cas9 group, suggesting liposome-CRISPR/Cas9 group and A10-liposome- CRISPR/Cas9 group displayed slightly stronger antitumor effect than the PBS group or free CRISPR/Cas9 group. Notably, no mortality was observed after 27-d treatment with the A10-liposome-CRISPR/Cas9 chimera, and no significant change in mouse body weight (Figure 6C) was observed compared to that of control, suggesting that this compound was non-toxic to the animals under the present conditions (Figure 6B, 6C).

To further examine the effects of the A10-liposome-CRISPR/Cas9 chimeras *in vivo*, As shown in (Figure 6D), xenografts derived from A10-liposome-PLK1 CRISPR/Cas9 chimeras grew more slowly in comparison with PBS, free CRISPR/Cas9, or A10-liposome-scrambled CRISPR/Cas9 chimeras. Histological examination of the tumor sections was performed. The tumor cells from mice treated with A10-liposome-PLK1 CRISPR/Cas9 chimeras, in contrast to those from mice treated with PBS, free CRISPR/Cas9, or A10-liposome-scrambled CRISPR/Cas9 chimeras, were vacuolated, extensively granulated, and showed evidence of necrosis (Figure 6F). Moreover, less epithelium was present in tumors from the A10-liposome- CRISPR/Cas9 chimera-treated mice. Conversely, the control tumors were dense and composed primarily of epithelium. Occasional pockets of necrosis were exhibited in the A10-liposome-scrambled CRISPR/Cas9 chimera-treated tumors, indicating that non-specific uptake and subsequent processing of the chimera might have occurred to a limited extent. However, because no substantial change in tumor volume was noted in the A10-liposome-scrambled CRISPR/Cas9 chimeras treated tumors (Figure 6A), we presumed that this uptake was an inefficient process.

### *In vivo* immunostimulatory effects of liposome- CRISPR/Cas9 chimeras

The potential immunostimulatory effects of the liposome-CRISPR/Cas9 chimera delivery system were determined by assessing levels of IFN-a and IL-12 in mouse serum by ELISA. As shown in Figure 6E, CRISPR/Cas9 alone or formulated with targeting liposomes induced a very mild production of inflammatory cytokines in athymic nude mice. Compared to the poly (I:C) positive control, all formulations had no significant immunogenicity. Furthermore, the addition of A10 to the targeting formulation induced lower levels of cytokines than the liposome CRISPR/Cas9 alone verifying that A10 conjugation reduces non-specific immunostimulatory effects, likely due to its ability to increase cell specificity.

## DISCUSSION

Targeting tumors is an attractive application of CRISPR/Cas9 system. Although various methods have been described for delivering CRISPR/Cas9, few reports have accomplished therapeutic success. One possible reason for the lack of success in *in vivo* studies is the nonspecific delivery effects. Therefore, cell type-specific delivery of gRNAs is a critical goal for the widespread applicability of this therapeutic technique due to both safety and cost considerations. a system which combines the efficient delivery and modified flexibility is likely to become the next focus of targeted delivery systems. Three considerations are important for developing this approach: the CRISPR/Cas9 designed to knockdown genes in a defined population of cells; the targeting moiety that specifically recognizes surface receptors expressed on the cell population of interest; and a low immunostimulatory system that links CRISPR/Cas9 to the targeting moiety with low cost, high ease and flexibility, optional modification, and suitable safety. Toward this end, using human prostate cancer as an example, we developed an aptamer-liposome based CRISPR/Cas9 chimera that recognized the PSMA receptor expressed on many prostate cancer cells.

For many potential therapeutic applications of chimeras that require systematic administration of therapeutic reagent, optimization of the formulation is necessary for full potential. The RNA aptamer A10 has been reported to have the capability to bind PSMA [26, 27], and therefore, our chimeras were designed with this RNA aptamer. This aptamer was modified with 2’-F and 3′-NH_*2*_ during the process of transcription to improve its stability and provide a convenient linkage*^21^*. Moreover, because terminal modification of RNAs with PEG increases the half-life of many oligonucleotide-based therapies, including RNA aptamers [28], PEGylation was also introduced into the chimera. Our results demonstrate that the binding of A10 is more competitive when modified with DSPE-PEG_*2000*_ (Figure 2D) than the A10 aptamer (Figure 2C). This suggests that appropriate modification can promote the function of aptamers without affecting chimera targeting (Figure 2) or knockdown (Figure 3).

The *in vitro* cell surface binding of different formulations demonstrated that cellular targeting of the chimeras was mediated by the interaction of the aptamer portion of the novel delivery system with PSMA on the cell surface (Figure 2B; Figure 3, lanes 8 and 9). The gene knockdown effect of different formulations showed high correlation with the CRISPR/Cas9-gRNA sequence and the intracellular delivery efficiency (Figure 3). Notably, in the presence of A10, liposome-CRISPR/Cas9 chimeras provided much greater delivery efficiency and higher gene knockdown activity than other formulations, demonstrating that the function of the delivery system was dependent on the interaction of A10 and PSMA.

Because many potential therapeutic applications of chimeras, including cancer therapy, require systemic administration of the therapeutic reagent, it is also necessary to optimize potency and specificity *in vivo*. The *in vivo* activity of the A10-liposome-CRISPR/Cas9 chimeras in promoting tumor regression was consistent with the *in vitro* observations, in which the A10-liposome- CRISPR/Cas9 chimeras showed significantly higher activity than other formulations (Figure 6A). We believe the enhanced activity of this novel delivery system is mainly due to the significantly improved tumor uptake (Figure 4F) and the specific binding to LNCap cells (Figure 2F). Formulations including free CRISPR/Cas9, non-targeted liposome-CRISPR/Cas9 chimeras and scrambled CRISPR/Cas9 in A10-liposome chimeras had little effect (Figure 6A), while A10-liposome-PLK1 CRISPR/Cas9 resulted in pronounced regression of tumors expressing PSMA in athymic mice after *i.v*. injection. The results suggest that significant gene knockingdown activity is highly dependent on the correct gRNA sequence and sufficient tumor delivery. Therefore, this delivery system is suitable for targeting tumors *in vivo* and could possibly be useful for therapeutic treatment of human prostate cancer in the future.

Previous studies have demonstrated a cytokine induction effect that is dependent on the CRISPR/Cas9 and the formulation [29, 30]. Our results (Figure 6E) showed little immune response of nude mice with different CRISPR/Cas9 formulations. Compared to the positive control, CRISPR/Cas9 alone or with all formulations had no significant immunogenicity, suggesting that the formulations we developed are safer than other cationic liposome. We also found that the targeted CRISPR/Cas9 delivery system, when formulated with A10, shows a lower immune response than non-targeted liposomes, which suggested that modification with A10 RNA aptamer has a potential ability to reduce the immunogenicity of the cationic liposome.

In principle, the current aptamer-liposome-CRISPR/Cas9 chimera approach can be applied to the development of reagents targeting many different cell types, provided that a cell-type-specific receptor exists and that an aptamer against the receptor can be selected. The flexibility of modification on the targeting moiety or liposome also should enable this system to develop CRISPR/Cas9-based therapeutics for a wide variety of diseases

## MATERIALS AND METHODS

### Cell culture

PC (prostate cancer) cell lines were obtained from American Type Culture Collection. LNCap cells (ATCC No. CRL-1740), PC-3 cells (ATCC No. CRL-1435) were grown in Roswell Park Memorial Institute medium 1640 (RPMI 1640, Invitrogen) supplemented with 10% FBS, 100 U/mL penicillin, and 100 U/mL streptomycin and were maintained at 37°C and 5% CO_*2*_. LNCap cells were shown to be PSMA positive by Western blotting [31] (Figure 2).

### Materials

DOTAP, cholesterol, and DSPE-PEG_*2000*_ were purchased from Avanti Polar Lipid, Inc. unless otherwise noted, all chemicals were purchased from Sigma-Aldrich, all cell culture products were purchased from Gibco BRL/Life Technologies, a division of Invitrogen, and all restriction enzymes were obtained from New England BioLabs (NEB).

### Design and cloning of PLK1-specific gRNA

gRNA expression plasmids were constructed according to manufacturer's protocol [32, 33] and detailed BLAST searches of the human and murine genomes were conducted to identify potential off-target binding of PLK1 gRNAs. To assess the utility of PLK1-targeting gRNAs, oligonucleotide were designed to target the complete genomic PLK1 (5′-CGGAGGCTCTGCTCGGATCG-3′). All oligonucleotides were synthesized and purified by Sangon Biotech Co (Shanghai, China). Briefly, to prepare a 100-bp double-stranded DNA insert fragment containing the target sequence (20 bp) and a protospacer-adjacent motif sequence, we used a set of oligonucleotides and generated the fragment using T4 PNK (NEB, Ipswich, MA, USA). The double-stranded DNA fragment was purified and inserted into the BbsI site of a gRNA cloning vector with T4 DNA ligase (NEB).

### Preparation of A10-liposome- CRISPR/Cas9 chimeras

### *In vitro* transcription of the A10 aptamer

Transcription was performed either with or without modified NTPs (2’F-dCTP, 2’F-dUTP and 3′NH_*2*_-dUTP) according to the instructions of the DuraScribe^®^ T7 Transcription Kit (Epicentre Biotechnologies). The 20 μL transcription reactions contained 2 μL 10 × T7 reaction buffer, 1 μL 100 mM ATP, 1 μL 100 mM GTP, 2 μL 50 mM 2’F-dCTP or CTP, 2 μL 50 mM 2’F-dUTP or 3′NH_*2*_-dUTP or UTP, 2 μL 100 mM DTT, 2 μL DuraScribe T7 RNA Enzyme Mix, and 1 μg A10 double-stranded PCR template.

### Conjugation of DSPE-PEG_2000_ and modified aptamer

Fifty microliters of DSPE-PEG_*2000*_-COOH (10 μg/μL in DNase/RNase-free water) was incubated with 100 μL of 800 mmol/L 1-(3-dimethylaminopropyl)- 3-ethylcarbodimide hydrochloride (EDC) and 100 μL of 200 mmol/L N-hydroxysuccinimide (NHS) for 15 min at room temperature with gentle stirring. Where indicated, the resulting NHS-activated DSPE-PEG_*2000*_ was covalently linked to 50 μL of 3′-NH_*2*_–modified A10 PSMA aptamer (1 μg/μL in DNase/RNase-free water). The aptamer-DSPE-PEG_*2000*_ bioconjugates were washed, resuspended, and preserved in suspension form in DNase/RNase-free water.

### Preparation of liposome-CRISPR/Cas9 chimeras

DOTAP and cholesterol were dissolved at a 3:1 molar ratio in a mixture of chloroform in a glass vial. The solvent was removed by reverse evaporation in a rotary evaporator at 25°C, 0.06 MPa. Five milliliters of sterile deionized water was added to the dried lipid film, and the mixture was allowed to swell overnight. The lipids were then sonicated in a bath-type sonicator for 5 min followed by extrusion through 450 and 220 nm membrane filters 5 times. This preparation was stored at 4°C before use. Novel liposome-CRISPR/Cas9 chimeras were composed of DOTAP/cholesterol liposome, protamine, and a mixture of gRNA and calf thymus DNA (0.75:1 weight ratio). To prepare the liposome-CRISPR/Cas9 chimeras, protamine (2 mg/mL), deionized water, and the gRNA/calf thymus DNA mixture (2 mg/mL) were combined in a 1.5 mL tube. The complex was allowed to stand at room temperature for 10 min before the addition of DOTAP/cholesterol liposome (300:1 molar ratio vs gRNA). The liposome- CRISPR/Cas9 chimeras were incubated at room temperature for another 10 min before further application.

### Conjugation of DSPE-PEG_2000_-A10 and liposome-CRISPR/Cas9 chimeras

The resultant mixture of DSPE-PEG_*2000*_-A10 bioconjugates (0.3:1 mole ratio vs DOTAP) and the liposome-CRISPR/Cas9 chimeras were incubated at 60°C for 1 h so that the ligand-anchors could rapidly insert into the outer liposome monolayer.

### Characterization of the A10-liposome-CRISPR/Cas9 chimeras

The particle size and zeta potential of the liposome-CRISPR/Cas9 chimeras were measured using the Nano Zetasizer (Malvern Instrument Ltd.). Average values were reported as the mean ± standard deviation. The liposome-gRNA chimeras were observed under transmission electron microscopy (TEM). Briefly, the liposomes were diluted ten-fold with distilled water and applied to 300 mesh, formvarcarbon-coated Cu grids. Chimeras were then negatively stained with 2% uranyl acetate (pH 4.8) for 30 s. Stained samples were characterized on a Philips CM120 TEM at a final magnification of 120,000 ×. Three grids were prepared for each sample and the grid openings were randomly selected and viewed.

### Cell-surface binding competition assays

LNCaP or PC-3 cells were used for cell-surface binding competitive experiments. Anti-PSMA 3C6 antibody (2 μg, R&D Systems Inc.), A10 aptamer, DSPE-PEG_*2000*_-A10, Mut-A10 aptamer or A10-liposome-CRISPR/Cas9 chimeras were used as antibody competitors. Cells were trypsinized, washed twice with 500 mL PBS, and fixed in 400 mL of FIX solution (PBS + 1% formaldehyde) for 20 min at 25°C. After washing with PBS, cells pellets were re-suspended in 1 × binding buffer (20 mM HEPES pH 7.4, 150 mM Nacl, 2 mM CaCl_*2*_, 0.01% BSA) containing 2 μg (1 μg/μL) PE modified anti-PSMA 3C6 antibody at 37°C for 20 min. Then different competitors were added to compete with PE modified anti-PSMA 3C6 antibody in PBS with 4% FBS pre-warmed at 37°C for 20 min. Cells were washed three times with PBS, fixed in 400 μL of FIX, and analyzed by flow cytometry.

In the 5-α-dihydrotestosterone (DHT, Sigma) treatment experiment, LNCap or PC-3 cells were grown in RPMI 1640 medium containing 5% charcoal-stripped serum for 24 h before the addition of DHT in RPMI 1640 medium containing 5% charcoal-stripped FBS for 48 h. And then competition assays were performed with different competitors described above. Cell surface competition was assessed by flow cytometry.

Total PSMA expression was analyzed by western blotting. Cells were trypsinized, and counted using a hemocytometer. Cell pellets were re-suspended in 1 × RIPA buffer (150 mM NaCl, 50 mM Tris-HCl pH 8.0, 1 mM EDTA, 1 % NP-40) containing 1 × protease and phosphatase inhibitor cocktails (Sigma) and incubated on ice for 20 min. Split products were boiled with 2 × protein loading buffer, and 15 μL of total protein from the supernatants were resolved on a 12.5 % SDS-PAGE gel. PSMA was detected using a 1:1000 dilution of an antibody specific to human PSMA (Abnova) and a 1:2000 dilution of secondary antibody (goat anti-mouse IgG) in blocking buffer. b-actin was detected using a 1:1000 dilution of an antibody specific to human β-actin (Cell Signaling Technology, Inc.) and a 1:2000 dilution of secondary antibody (goat anti-mouse IgG) in blocking buffer.

### Gene knockdown assay

LNCap or PC-3 cells were seeded in 6-well plates at 60% confluency. Cells were transfected with CRISPR/Cas9 on day 3 using Lipofectamine-2000 (Invitrogen) following the manufacturer's recommendations. In parallel, cells were treated with free CRISPR/Cas9, liposome-CRISPR/Cas9 chimeras, liposome-CRISPR/Cas9 chimeras (with protamine and calf thymus), A10-liposome-CRISPR/Cas9 chimeras or A10-liposome- CRISPR/Cas9 chimeras (with protamine and calf thymus). To further verify that silencing by liposome- CRISPR/Cas9 chimeras is dependent on PSMA, LNCap or PC-3 cells were incubated with 2 nM DHT for 48 h before the addition of chimeras. Cells were collected on day 5 for analysis. Gene knowkdown was assessed by RT-PCR of mRNA (50 ng) from cells treated with the various CRISPR/Cas9 or chimeras using the One-Step RT-PCR Kit (Tiangen, Beijing). All reactions were done in a 50-mL volume in triplicate. PCR parameters were as follows: 50°C for 30 min, 5 min of Taq activation at 95°C, followed by 45 cycles of 95°C × 30 s, 57°C × 30 s, 72°C × 30 s. Standard curves were generated, and the relative amount of target gene mRNA was normalized to GAPDH mRNA. Specificity was verified by agarose gel electrophoresis.

### Cellular uptake study

LNCap cells (2.25 × 10*^5^* per well) were seeded in 6-well plates (Corning Inc.) and allowed to attach and grow overnight at 37°C and 5% CO_*2*_. Cells were washed with PBS 3 times and incubated with various concentrations of FAM labeled siRNAs, Lipofectamine-2000-FAM labeled CRISPR/Cas9 chimeras, liposome-CRISPR/Cas9 chimeras, liposome-CRISPR/Cas9 chimeras with protamine and calf thymus DNA, or A10-liposome-CRISPR/Cas9 chimeras with protamine and calf thymus DNA for 4 hr at 37°C. Treatments were done in triplicate. The cells were then washed with PBS 3 times and lysed with 1 mL of 0.1% Triton-X-100 in PBS. The cells were collected and measured for the fluorescence intensity at λem = 562 nm and λex = 494 nm by microscopy using a Nikon eclipse TE 300 Confocal microscope.

### Cell apoptosis assay

The apoptosis of LNCap cells in the presence or absence of different chimeras, or without any treatment as a blank control, was evaluated by flow cytometry. the cells were collected and stained with the Annexin V-FITC apoptosis detection kit (KeyGEN, Nanjing, China) according to the manufacturer's instructions, and were immediately analyzed via the FACScan flow cytometer with 10,000 events collected (Ex: 488 nm; Em: 530 nm).

### Cell viability

The viability of LNCap cells in the presence or absence of different chimeras, or without any treatment as a blank control, was evaluated by 3-[4,5-dimethylthiazol-2-yl]-2,5-diphenyl- tetrazoliumbromide (MTT) assay over a period of 24 h. LNCap cells were seeded at a density of 5,000 cells/well (200 μL) in 96-well flat-bottomed microtiter plates over-night. Cells were treated with PBS once and incubated with 400 nM free siRNA, Lipofectamine-2000, liposomes, liposomes with protamine and calf thymus DNA, A10-liposomes, A10-liposomes with protamine and calf thymus, CRISPR/Cas9-Lipofectamine-2000 chimeras, liposome-CRISPR/Cas9 chimeras, liposome-CRISPR/Cas9 chimeras (with protamine and calf thymus DNA), or A10-liposome-CRISPR/Cas9 chimeras (with protamine and calf thymus DNA) for 24 h at 37°C, and then washed 3 times with PBS. 20 μL of MTT solution (5 mg/mL in PBS) was then added to each well and the cells were incubated further for 1 hr at 37°C. The media were removed and the cells were dissolved in DMSO. Ultraviolet absorbance at 555 nm was measured in Thermo Electron Corporation Multiskan MK3 (Thermo Fisher Scientific Inc.). The data are expressed as the percent of viable cells compared to untreated control cells.

### Animal experiments

Male athymic nude mice (nu/nu) of age 6–8 weeks were obtained from BioDure Technology (Beijing, China) and maintained in a sterile environment. The First Affiliated Hospital of Xi’an Jiaotong University Institutional Animal Licensing Committee approved the animal experiments undertaken, and the research protocol was in accordance with the First Affiliated Hospital of Xi’an Jiaotong University institutional guidelines for the Animal Care and Use Committee. Mice were inoculated with LNCap cells and randomly divided into five groups of nine mice per treatment group as follows: no treatment (PBS); treatment with free CRISPR/Cas9 (40 μg gRNA + 40 μg Cas9); treatment with A10-liposomes-Scrambled CRISPR/Cas9 chimeras (40 μg A10-liposomes-Scrambled gRNA+ 40 μg Cas9); treatment with liposomes-CRISPR/Cas9 chimeras (40 μg liposomes-gRNA + 40 μg Cas9); treatment with A10-liposomes- CRISPR/Cas9 chimeras (40 μg A10-liposomes-gRNA + 40 μg Cas9). Tumors were measured every other day with calipers in two dimensions. The following formula was used to calculate tumor volume: V_*T*_ = W × L × L × 0.5 (W, the longest dimension; L, the shortest dimension). The growth curves are plotted as the mean tumor volume ± S.E.M. The animals were sacrificed 3 d after the last treatment, and the tumors were excised and formalin fithe for immunohistochemistry. Slides of serial sections were stained with hematoxylin and eosin (H&E).

### Cytokine induction assay

Male athymic nude mice (nu/nu) were injected *i.v*. with PBS, free CRISPR/Cas9 (40 μg gRNA + 40 μg Cas9), A10-liposomes-Scrambled CRISPR/Cas9 chimeras (40 μg A10-liposomes-Scrambled gRNA + 40 μg Cas9), liposomes- CRISPR/Cas9 chimeras (40 μg liposomes-gRNA + 40 μg Cas9), A10-liposomes-CRISPR/Cas9 chimeras (40 μg A10-liposomes-gRNA + 40 μg Cas9), or poly (I:C) (25 μg). Just prior to use, the poly (I:C) stock was diluted 1:5 in PBS to provide a 500 μg/mL working solution. Two hours after the injections, blood samples were collected from the tail artery and incubated at room temperature for 0.5 h for coagulation. Serum was obtained by centrifuging the clotted blood at 16,000 rpm for 20–40 min. Cytokine levels were determined using ELISA kits for IL-12 and IFN-α (BD Biosciences, San Diego, CA).

### Statistical analysis

Statistical analysis was conducted using one-way ANOVA, Mann Whitney. *P* < 0.05 was considered to indicate a significant difference. The survival rate was analyzed by the chi-square test.

## CONCLUSIONS

An aptamer-liposome-CRISPR/Cas9 delivery chimera-based approach was designed that combines efficient delivery and modified flexibility. The aptamer, modified A10 in the chimeras mediates cell type-specific binding to human PSMA, a cell-surface receptor expressing in prostate cancer cells, whereas, the therapeutic CRISPR/Cas9 target PLK1. The aptamer-liposome-CRISPR/Cas9 chimeras are demonstrated not only had a significant cell-type specificity in binding and a remarkable gene silencing effect *in vitro*, but also a conspicuous tumor regression *in vivo*, while showed a lower immune response. The flexibility of modification on the targeting moiety or liposome we described here provides a universal means of cell type-specific CRISPR/Cas9 delivery, which is a critical goal for the widespread therapeutic applicability of CRISPR/Cas9 or other nucleic acid drugs.
